# Identification of miR-26 as a key mediator of estrogen stimulated cell proliferation by targeting CHD1, GREB1 and KPNA2

**DOI:** 10.1186/bcr3644

**Published:** 2014-04-15

**Authors:** Sheng Tan, Keshuo Ding, Rui Li, Weijie Zhang, Gaopeng Li, Xiangjun Kong, Pengxu Qian, Peter E Lobie, Tao Zhu

**Affiliations:** 1 Laboratory of Molecular Tumor Pathology, School of Life Sciences, University of Science and Technology of China, Hefei 230027, China; 2 Cancer Science Institute of Singapore, National University of Singapore, Centre for Translational Medicine MD6, #11-01 K, 14 Medical Drive, Singapore 117599, Singapore; 3 National Cancer Institute of Singapore, National University Health System, Singapore, Singapore; 4 Hefei National Laboratory for Physical Sciences at Microscale and School of Life Sciences, University of Science and Technology of China, Hefei, Anhui 230027, People’s Republic of China

## Abstract

**Introduction:**

Estrogen signaling is pivotal in the progression of estrogen receptor positive breast cancer primarily by the regulation of cell survival and proliferation. Micro (mi)RNAs have been demonstrated to be regulated by estrogen to mediate estrogenic effects. Herein, we determined the role of estrogen regulated miR-26 and its underlying molecular mechanisms associated with estrogen receptor (ER)+ breast cancer proliferation.

**Methods:**

The expression of miR-26a and miR-26b was evaluated by real-time quantitative (RT)-PCR. The expression of miR-26a or miR-26b was modulated in ER+ breast cancer cells (MCF-7 and T47D) and tumor cell growth *in vitro* and an *in vivo* xenograft model was determined. Bioinformatics analyses were utilized to screen for estrogen responsive genes, which were also predicted to be targeted by miR-26. Luciferase reporter assays were performed to confirm miR-26 regulation of the 3' UTR of target genes. The levels of miR-26 target genes (*CHD1*, *GREB1* and *KPNA2*) were evaluated by western blotting and immunohistochemistry.

**Results:**

Estrogen reduced the expression of miR-26a and miR-26b in ER+ breast cancer cells. Forced expression of miR-26a or miR-26b significantly inhibited the estrogen stimulated growth of ER+ breast cancer cells and tumor growth in xenograft models, whereas miR-26a/b depletion increased the growth of ER+ breast cancer cells in the absence of estrogen treatment. Screening of estrogen responsive genes, which were also predicted to be targeted by miR-26, identified *GREB1* and nine other genes (*AGPAT5*, *AMMECR1*, *CHD1*, *ERLIN1*, *HSPA8*, *KPNA2*, *MREG, NARG1,* and *PLOD2*). Further verification has identified nine genes (*AGPAT5, CHD1, ERLIN1, GREB1, HSPA8, KPNA2, MREG, NARG1* and *PLOD2*) which were directly targeted by miR-26 via their 3′ UTR. Functional screening suggested only three estrogen regulated miR-26 target genes (*CHD1, GREB1* and *KPNA2*) were involved in the regulation of estrogen promoted cell proliferation. Depletion of either CHD1, GREB1 or KPNA2 significantly abrogated the enhanced growth of ER+ breast cancer cells due to miR-26 depletion. We further demonstrated that estrogen stimulated c-MYC expression was both sufficient and necessary for the diminished expression of miR-26a and miR-26b.

**Conclusions:**

We have identified a novel estrogen/MYC/miR-26 axis that mediates estrogen stimulated cell growth via CHD1, GREB1 and KPNA2.

## Introduction

Breast cancer is the most common malignant disease in women. However, the molecular pathogenesis of breast cancer remains poorly defined due to its heterogeneity [1]. Approximately 70% of human breast cancer is estrogen receptor (ER)-alpha-positive and estrogen signaling plays a pivotal role in both the pathogenesis and the progression of ER+ breast cancer [2]. One of pathological effects of estrogen in hormone-responsive breast cancer is to promote proliferation and tumor growth [3]. In postmenopausal women with early-stage ER+ breast cancer, anti-estrogen therapy is utilized as an effective adjuvant treatment. However, many patients whose tumors respond to anti-estrogen therapy eventually develop resistance and tumor recurrence [4]. An improved understanding of the molecular basis of estrogen action and the development of new strategies to improve the efficacy of anti-estrogens are therefore required.

MicroRNAs (miRNAs) are noncoding RNAs that range in size from 20 to 25 nucleotides and promote mRNA degradation and/or inhibition of translation by base pairing with the 3′ untranslated region (UTR) of target mRNAs [5]. Multiple recent reports have established miRNAs as important for the initiation, promotion and progression of various human cancers [6,7]. miRNAs have been identified to function in both oncogenic and tumor suppresser roles [6,7]. The therapeutic manipulation of miRNAs may present an attractive clinical strategy because one miRNA could potentially regulate the coordinated expression of hundreds of different genes [8,9]. Various recent reports have observed that miRNAs are often deregulated in breast cancer [10,11] and that several deregulated miRNAs (for example, let-7, miR-21, miR-125b, miR-221 and miR-222) may possess important roles in breast cancer progression by contributing to cell proliferation, survival and metastasis [12-17]. The expression of some miRNAs has also been demonstrated to be regulated by estrogen in an ER-dependent manner [18-20]. The identification of the targets of estrogen-regulated miRNAs is critical to understand function of the miRNAs and their underlying molecular mechanisms associated with ER+ breast cancer progression.

In this study, we report that the expression of miR-26a and miR-26b was decreased by estrogen stimulation and that forced expression of miR-26a or miR-26b abrogated estrogen-stimulated breast cancer cell growth both *in vitro* and *in vivo*. CHD1, GREB1 and KPNA2 were identified as estrogen-regulated direct targets of miR-26a/b. The expression of CHD1, GREB1 or KPNA2 was required for estrogen-stimulated breast cancer cell growth. We further demonstrated that estrogen-regulated c-MYC expression was required for the suppression of miR-26a and miR-26b levels. Methods to enhance miR-26 expression may thus be considered as an adjuvant therapeutic strategy for patients with ER + breast cancer.

## Methods

### Cell culture

MCF-7, T47D, MDA-MB-231 and BT549 cells were purchased from American Type Culture Collection and were cultured in the recommended conditions. All cells were maintained in a humidified incubator at 37°C and 5% carbon dioxide. For 17β-estradiol (E2) stimulation experiments, cells were cultured for at least 3 days in phenol red-free RPMI 1640 with 5% dextran-coated charcoal-treated serum before E2 (10^−8^ mol/l) treatment.

### Plasmid construction

To stably express miR-26a or miR-26b in MCF-7 cells, the retroviral vector pBABE-Puro-miR-26a or pBABE-Puro-miR-26b was constructed. The genome segment encompassing the mature miR-26a sequence was as described previously [21] or the 77 base pair DNA fragment corresponding to pre-miR-26b was amplified from human genomic DNA and then cloned into the pBABE-Puro plasmid. To stably deplete miR-26a or miR-26b in MCF-7 and T47D cells, we generated miR-26a and miR-26b sponge elements. We introduced nine copies of CCTATCCACCATTACTTGAA complementary sequences to miR-26a and miR-26b, each with mismatches at positions 11 to 13 for improved stability, into pBABE-Puro expression vector [22,23].

### RNA oligonucleotides and transfection

miRNAs and small interfering RNAs (siRNAs) were synthesized by GenePharma (Shanghai, China). miRNA mimics are synthetic duplexes representing mature miRNAs. siRNA and miRNA transfection was performed using lip2000 (QIAGEN). 20 nmol/l siRNA or miRNA was used for transfection in serum-free medium. Total RNA and protein were prepared 48 to 72 hours after transfection and further used for quantitative polymerase chain reaction (PCR) or western blot analysis.

### Quantitative analysis of miRNAs and mRNAs

Total RNA and miRNA were extracted from cultured cells or clinical samples of breast cancer using the miRVana miRNA Isolation Kit (Ambion) according to the manufacturer’s protocols. The TaqMan stem-loop real-time (RT)-PCR approach was used to assess the expression of miRNAs with kits from Applied Biosystems. For quantitative analysis of mRNA expression, 100 to 200 ng total RNA was used for synthesis of random-primed single-stranded cDNA using the Primescript RT reagent kit (TaKaRa) and cDNA was subjected to quantitative PCR using SYBR green Master MIX (Applied Biosystem). The relative amount of gene transcripts were normalized to glyceraldehyde 3-phosphate dehydrogenase. Three independent experiments were each performed in triplicate.

### Protein extraction and western blot

Cells were lysed using cell lysis buffer (Cell Signaling) and a protein concentration determined with the BCA Protein Assay kit (Pierce). Equal amounts of total proteins were separated in 10% SDS polyacrylamide gels and transferred to polyvinylidene difluoride membranes (Bio-Rad). Membranes were blocked for 1 hour with 1% bovine serum albumin in Tris-buffered saline containing 0.05% Tween 20, incubated overnight with primary antibody, washed and incubated with secondary antibody, and visualized by chemiluminescence. The antibodies used were as follows: CHD1 (Millipore), GREB1 (Abcam), KPNA2 (Abcam) and β-actin (Abcam).

### Luciferase reporter assay

The full-length 3′ UTR of the genes were amplified and cloned downstream of *Renilla luciferase* in a psiCHECK2 vector (Promega). Cells plated on 24-well plates were transfected with 100 ng plasmid and 200 nmol/l miR-26a, miR-26b mimics or negative control. After 48 hours, cells were lysed and assayed with Dual Luciferase Assay (Promega) according to the manufacturer’s instructions. Three independent experiments were performed in triplicate.

### MTT assay

Cells (10^3^ per well) were plated in 96-well plates in a final volume of 100 μl. Twenty-four hours after plating, 10 pmol miRNA mimics, siRNAs or negative control oligonucleotides were transfected into the cells with lip2000 (QIAGEN). The MTT assay was performed at 24, 48, 72 and 96 hours as described previously [24].

### Tumor xenograft in nude mice

MCF-7-NC, MCF-7-miR-26a or MCF-7-miR-26b cells (3 × 10^6^ cells per site) were injected into the mammary fat pad of 4-week-old BALBc nu/nu mice (Shanghai Slaccas). Long-release E2 pellets (Innovative Research of America) were implanted the day before inoculation.

### In vivo protocol approval

Research protocols were designed and conducted in accordance with the guidelines set by the Institutional Animal Care and Use Committee, University of Science and Technology of China (USTCACUC1301016).

## Results

### Decreased expression of miR-26 is required for estrogen-promoted cell proliferation

**Figure 1:**
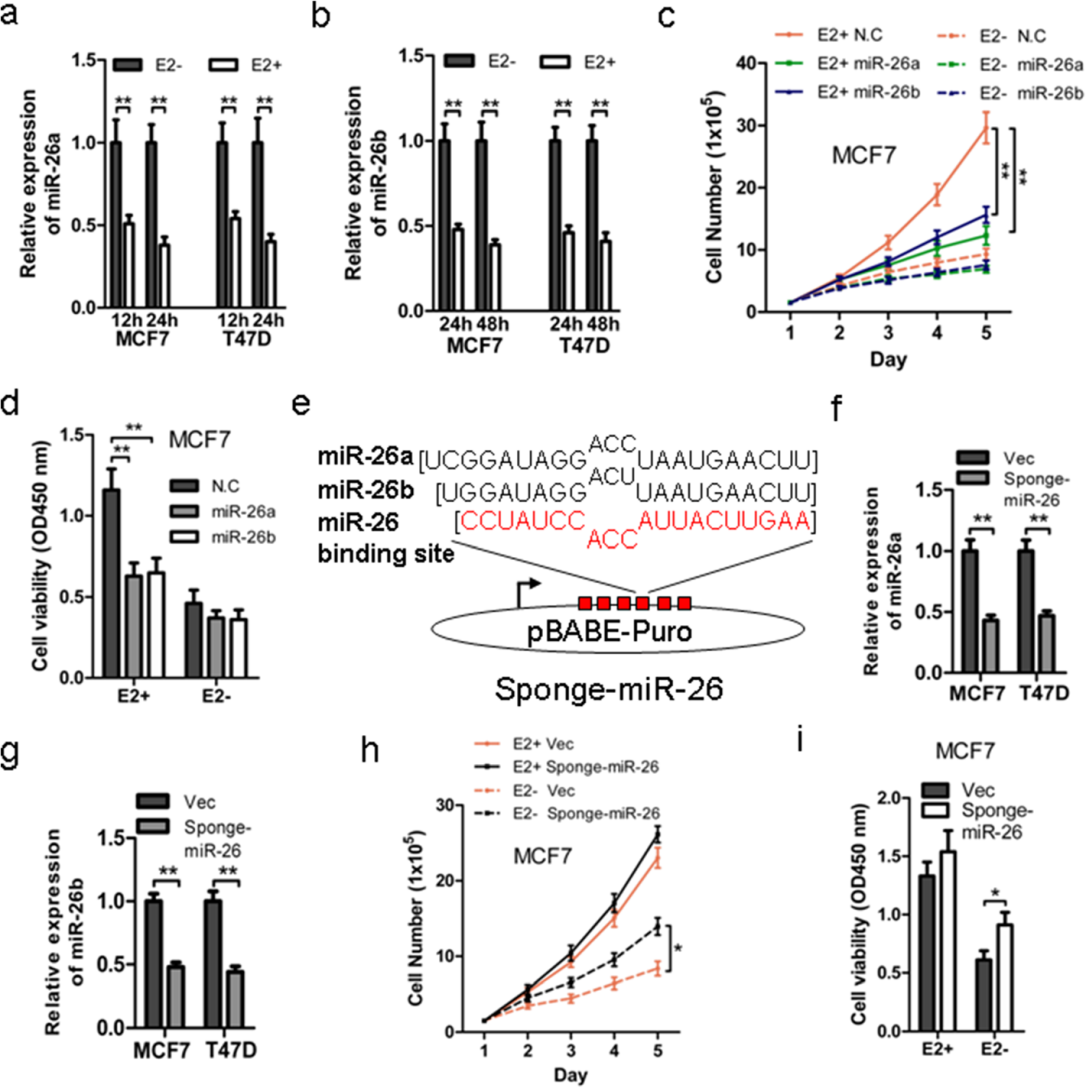
**miR-26 inhibits breast cancer cell growth. (a, b)** Effect of 17β-estradiol (E2) on miR-26a and miR-26b expression. MCF-7 and T47D were treated with 10 nmol/l E2 in phenol red-free medium containing 5% charcoal-stripped fetal bovine serum and microRNAs (miRNAs) from a triplicate sample were isolated at indicated time points. The miRNA level was measured by TaqMan stem-loop quantitative real-time polymerase chain reaction (qRT-PCR). U6 was used as an internal control. Relative expression level of (a) miR-26a and (b) miR-26b. **(c)** MCF-7 cells after 2 days of E2 deprivation were transfected with NC, miR-26a or miR-26b mimics and 1 day later were seeded in six-well plates with or without 10 nmol/l E2. At the indicated time points after transfection, cells were trypsinized and total cell numbers were counted using Trypan blue. **(d)** MCF-7 cells after 2 days of E2 deprival were transfected with NC, miR-26a or miR-26b mimics and 1 day later were seeded in 96-well plates with or without 10 nmol/l E2. At 72 hours after plating, the MTT assay was performed to determine the proliferation (viability) of MCF-7 cells. **(e)** Sequences of mature miR-26a/b, and one repeat of sponge. **(f, g)** Relative expression levels of miR-26a and miR-26b between MCF-7-Vec, MCF-7-Sponge-miR-26 or T47D-Vec, T47D-Sponge-miR-26 were measured by TaqMan stem-loop qRT-PCR. **(h)** MCF-7-Vec and MCF-7-Sponge-miR-26 cells after 3 days of E2 deprivation were seeded in six-well plates with or without 10 nmol/l E2. At the indicated time points, MCF-7-Vec and MCF-7-Sponge-miR-26 cells were trypsinized and total cell numbers were counted using Trypan blue. **(i)** MCF-7-Vec and MCF-7-Sponge-miR-26 cells after 3 days of E2 deprivation were seeded in 96-well plates with or without 10 nmol/l E2. At 96 hours after plating, the MTT assay was performed to determine the proliferation of MCF-7-Vec and MCF-7-Sponge-miR-26 cells. *P < 0.05. **P < 0.01.

We have previously screened estrogen-regulated miRNAs using miRNA microarray profiling [17]. In addition to let-7 g, which was downregulated by estrogen, miR-26a and miR-26b were among other estrogen-regulated miRNAs in ER-alpha-positive MCF-7 cells (Figure S1 in Additional file 1). ER+ MCF-7 and T47D breast cancer cells were used to verify estrogen regulation of miR-26a and miR-26b expression using TaqMan stem-loop RT-PCR analysis. The miRNA expression values were normalized to U6. The expression of miR-26a and miR-26b significantly decreased after both 12 and 24 hours treatment with 10^−8^ mol/l E2 in MCF-7 and T47D cells with prior estrogen deprivation (Figure 1a,b).

We next determined whether miR-26a/b modulated growth of ER+ breast cancer cells. MCF-7 and T47D cells were thus transfected with negative control, miR-26a mimics or miR-26b mimics. Forced expression of miR-26a or miR-26b inhibited the estrogen-simulated increase in cell viability and total cell number in MCF-7 (Figure 1c,d) and T47D cells (Figure S2 in Additional file 1) when compared with control oligonucleotides. However, forced expression of miR-26a or miR-26b did not significantly affect either cell viability or total cell number in both types of cells when cultured in estrogen-deprived conditions (Figure 1c,d; Figure S2 in Additional file 1) compared with control oligonucleotides. Moreover, forced expression of miR-26a or miR-26b did not significantly affect either cell viability or total cell number of the ER-negative MDA-MB-231 and BT549 cell lines (Figure S2 in Additional file 1). Hence it is apparent that forced expression of miR-26a or miR-26b abrogated the proliferative effects of E2 in ER+ breast cancer cells.

We further constructed a miR-26a and miR-26b dual specific sponge, namely Sponge-miR-26, to deplete both endogenous miR-26a and miR-26b (Figure 1e) and to determine whether reduced miR-26a/b levels were required for estrogen promotion of mammary carcinoma cell proliferation. MCF-7-Sponge-miR-26 and T47D-Sponge-miR-26 as well as their respective control cells (MCF-7-Vec and T47D-Vec) were generated by forced expression of Sponge-miR-26 or empty vector. Forced expression of Sponge-miR-26 significantly reduced the expression of both miR-26a and miR-26b (Figure 1f,g). miR-26 depletion in MCF-7 and T47D cells resulted in increased cell growth under estrogen-deprived conditions (Figure 1h,i; Figure S2 in Additional file 1). However, no significant differences in estrogen-simulated cell viability or total cell number were observed upon miR-26 depletion in those ER+ cells. Hence, it is apparent that estrogen repression of miR-26 expression is required for estrogenic effects in ER+ breast cancer cells.

### miR-26 expression suppressed breast cancer growth in nude mice

**Figure 2:**
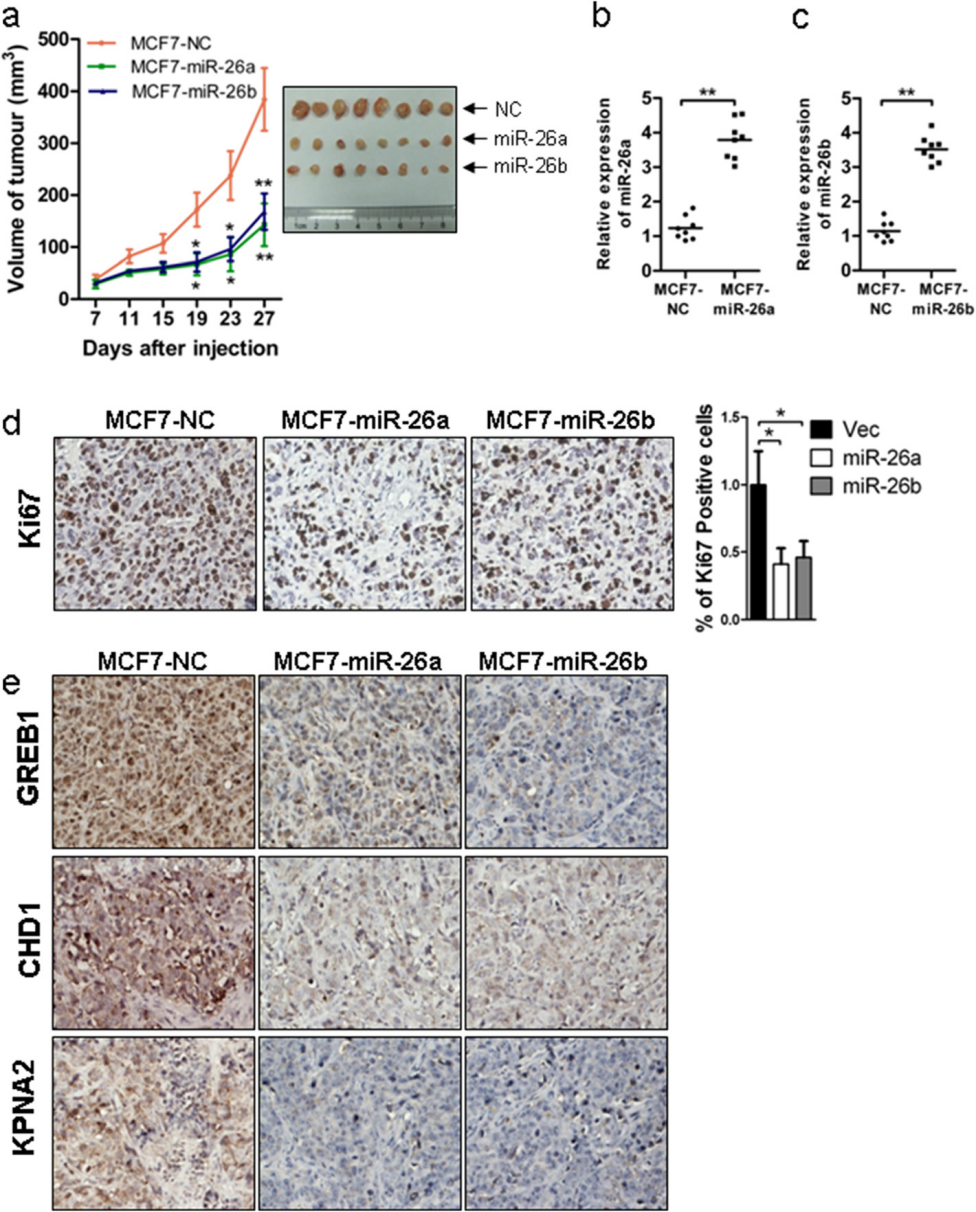
**miR-26 inhibits breast tumor growth *in vivo. *(a)** MCF-7-NC, MCF-7-miR-26a and MCF-7-miR-26b cells mixed with matrigel were transplanted into the mammary fat pad of immunodeficient mice supplemented with 17β-estradiol (E2), and tumor sizes were measured every 4 days until day 27 and the tumors were harvested. **(b, c)** The relative expression levels of miR-26a/b between tumors derived from MCF-7-NC and tumors generated from MCF-7-miR-26a respectively were quantified by TaqMan stem-loop quantitative real-time polymerase chain reaction. U6 was used as an internal control. **(d)** Tumors derived from MCF-7-miR-26a/b cells showed a lower level of Ki-67 protein than tumors derived from MCF-7-NC cells. **(e)** Immunohistochemistry analysis of GREB1, CHD1 and KPNA2 expression for tumor sections. *P < 0.05. **P < 0.01.

We further determined whether miRNA-26 expression could suppress breast cancer cell growth *in vivo*. MCF-7-miR-26a, MCF-7-miR-26b and cognate control cells were injected subcutaneously into female BALB/c nude mice in the presence of exogenous estrogen supplementation. Inoculation of MCF-7-miR-26a or MCF-7-miR-26b cells resulted in the formation of slower growing and significantly smaller tumors compared with tumors derived from MCF-7-NC inoculated mice (Figure 2a). The miR-26a and miR-26b expression levels were quantified in tumors derived from MCF-7-miR-26a or MCF-7-miR-26b cells (Figure 2b,c). A significantly lower proportion of proliferative Ki67+ tumor cells were observed by immunohistochemical analysis in tumor sections derived from MCF-7-miR-26a or MCF-7-miR-26b cells compared with the control cells (Figure 2d).

### Identification of targets of miR-26 involved in estrogen-promoted cell growth

**Figure 3:**
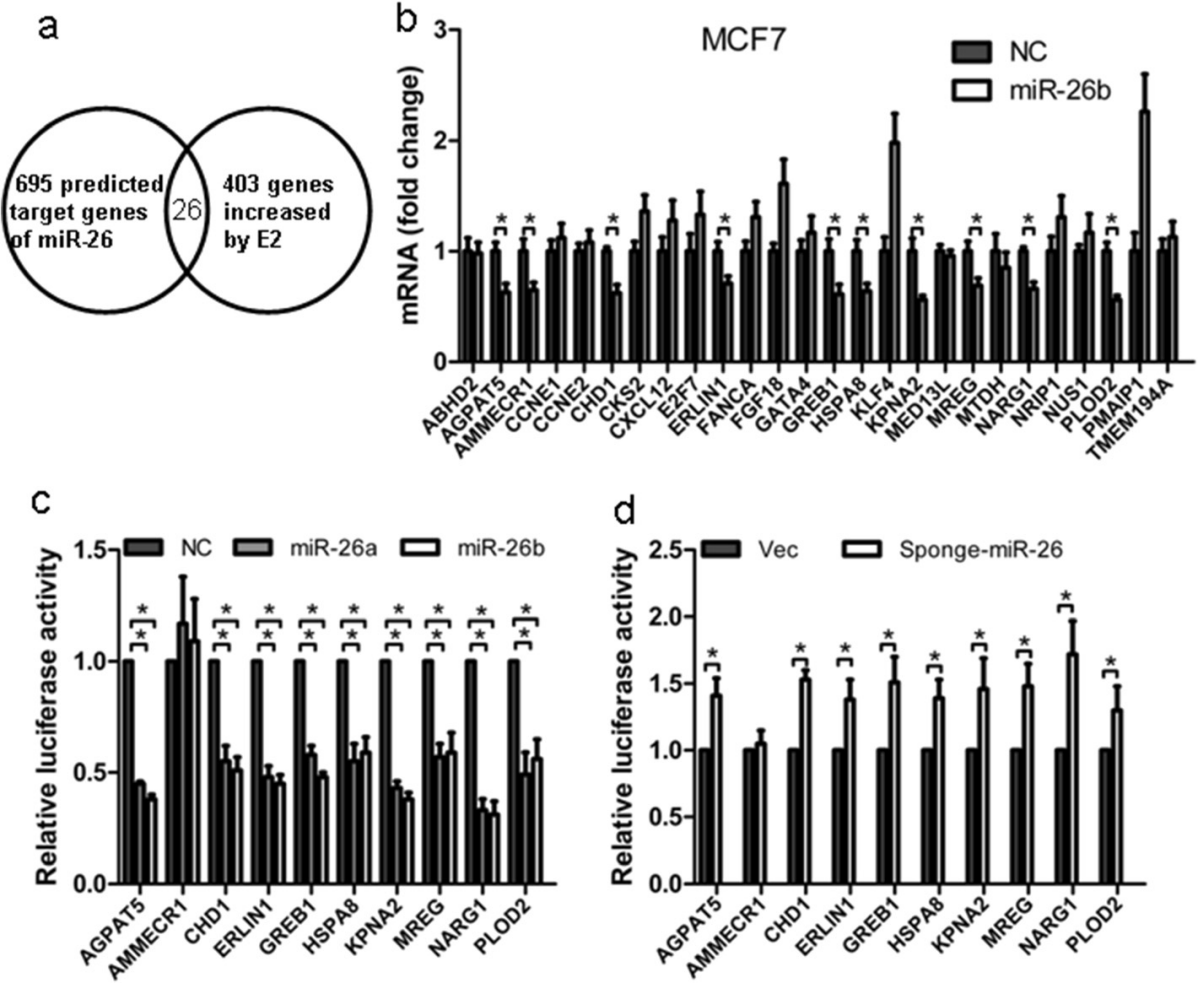
**Regulation of gene expression by miR-26. (a)** Venn diagram showing the number of genes identified by prediction methods compared with differential expression in 17β-estradiol (E2)-treated MCF-7 cells. **(b)** Ectopic expression of miR-26b in MCF-7 cells decreased mRNA levels of the indicated 10 genes by quantitative real-time polymerase chain reaction. Glyceraldehyde 3-phosphate dehydrogenase was used as an internal control. **(c)** Luciferase reporter assays in T47D cells, with co-transfection of the wild-type 3′ utranslated region (UTR) and miRNA as indicated. **(d)** Luciferase reporter assays in T47D cells, with co-transfection of wild-type 3′ UTR and miRNA sponge as indicated. *P < 0.05. **P < 0.01.

To identify downstream targets of miR-26, we performed bioinformatics analysis using three algorithms that predict the mRNA targets of a particular miRNA: TargetScan, PicTar and miRanda. A total of 695 putative target genes of miR-26 were predicted by at least one of the three algorithms (Figure 3a). Four independent gene expression datasets [25-28] profiling estrogen-regulated genes in MCF-7 cells were also employed for screening estrogen-regulated genes. A total of 403 genes were upregulated in E2-treated MCF-7 cells compared with the control cells. The overlap between the putative target genes of miR-26 and E2-upregulated genes yielded a set of 26 genes, which were considered as candidate target genes utilized by miR-26 to mediate estrogen-stimulated cellular effects.

To determine which of the 26 genes could be actually regulated by miR-26, RT-PCR analysis was performed to examine their expression with forced expression of miR-26b in MCF-7 or T47D cells. The mRNA levels of 10 genes (*AGPAT5*, *AMMECR1*, *CHD1*, *ERLIN1*, *GREB1*, *HSPA8*, *KPNA2*, *MREG*, *NARG1* and *PLOD2*) significantly decreased following the forced expression of miR-26b (Figure 3b; Figure S3 in Additional file 1). Moreover, we further verified that the expression of the 10 genes at the mRNA level was upregulated following estrogen treatment (Figure S4 in Additional file 1), suggesting that those 10 genes could be regulated by both estrogen and miR-26.

We further cloned the 3′ UTR of all 10 genes and generated fusions to a luciferase reporter gene. Forced expression of miR-26a or miR-26b mimics significantly reduced the activity of luciferase reporter gene fused to the 3′ UTR of all the candidate target genes (except *AMMECR1*) by more than 40% (Figure 3c). These data suggest that miR-26a and miR-26b could directly target nine genes via their respective 3′ UTR. Consistently, depletion of miR-26a/b by miR-26 sponge could increase the activity of luciferase reporter genes fused to the 3′ UTR of the same cohort of nine genes (*AGPAT5*, *CHD1*, *ERLIN1*, *GREB1*, *HSPA8*, *KPNA2*, *MREG*, *NARG1* and *PLOD2*) by more than 30% (Figure 3d). The activity of a luciferase reporter gene containing the AMMECR1 3′ UTR exhibited minimal change upon depletion of endogenous miR-26 (Figure 3d).

### CHD1, GREB1 and KPNA2 as critical mediators of miR-26 elicited cell growth

**Figure 4:**
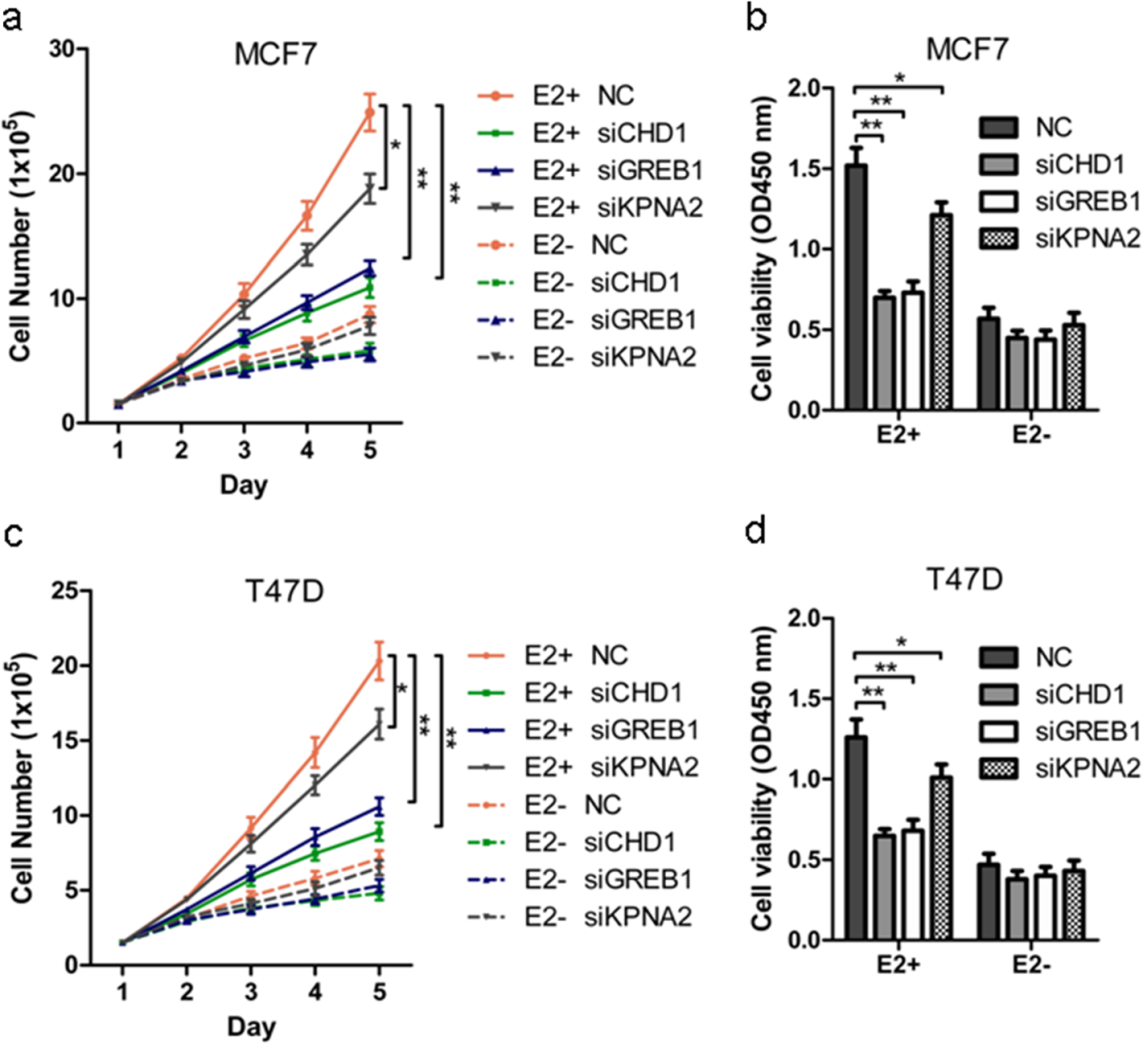
**Small interfering RNA-mediated depletion of CHD1, GREB1 or KPNA2 expression recapitulates the tumor suppressive functions of miR-26. (a, c)** MCF-7 or T47D cells deprived of estrogen for 2 days were infected with NC or small interfering RNA (siRNA) and 1 day later were seeded in six-well plates with or without 10 nmol/l 17β-estradiol (E2). At the indicated time points after transfection, cells were trypsinized and total cell numbers were counted using Trypan blue. **(b, d)** MCF-7 or T47D cells deprived of estrogen for 2 days were infected with NC or siRNA and 1 day later were seeded in 96-well plates with or without 10 nmol/l E2. At 72 hours after plating, the MTT assay was performed to determine the proliferation of MCF-7 and T47D cells. *P < 0.05. **P < 0.01.

Given that miR-26 negatively regulated ER+ breast cancer cell proliferation, we wished to determine which of the identified targets of miR-26 actually mediated miR-26-influenced cell growth. MCF-7 and T47D cells were thus transfected with siRNAs against these nine genes and the efficacy of siRNAs verified by RT-PCR (Figure S5 in Additional file 1). Interestingly silencing of *CHD1*, *GREB1* or *KPNA2* but not the remainder of the miR-26-regulated target genes significantly decreased E2-promoted growth of MCF-7 and T47D cells (Figure 4). In addition, silencing of the other six genes did not significantly affect the growth of MCF-7 and T47D cells in the presence or absence of estrogen (Figure S6 in Additional file 1), suggesting that the regulation of *AGPAT5*, *ERLIN1*, *HSPA8*, *MREG*, *NARG1* or *PLOD2* by miR-26a and miR-26b may be used for other cellular functions in breast cancer cells.

### CHD1, GREB1 and KPNA2 are the direct targets of miR-26

**Figure 5:**
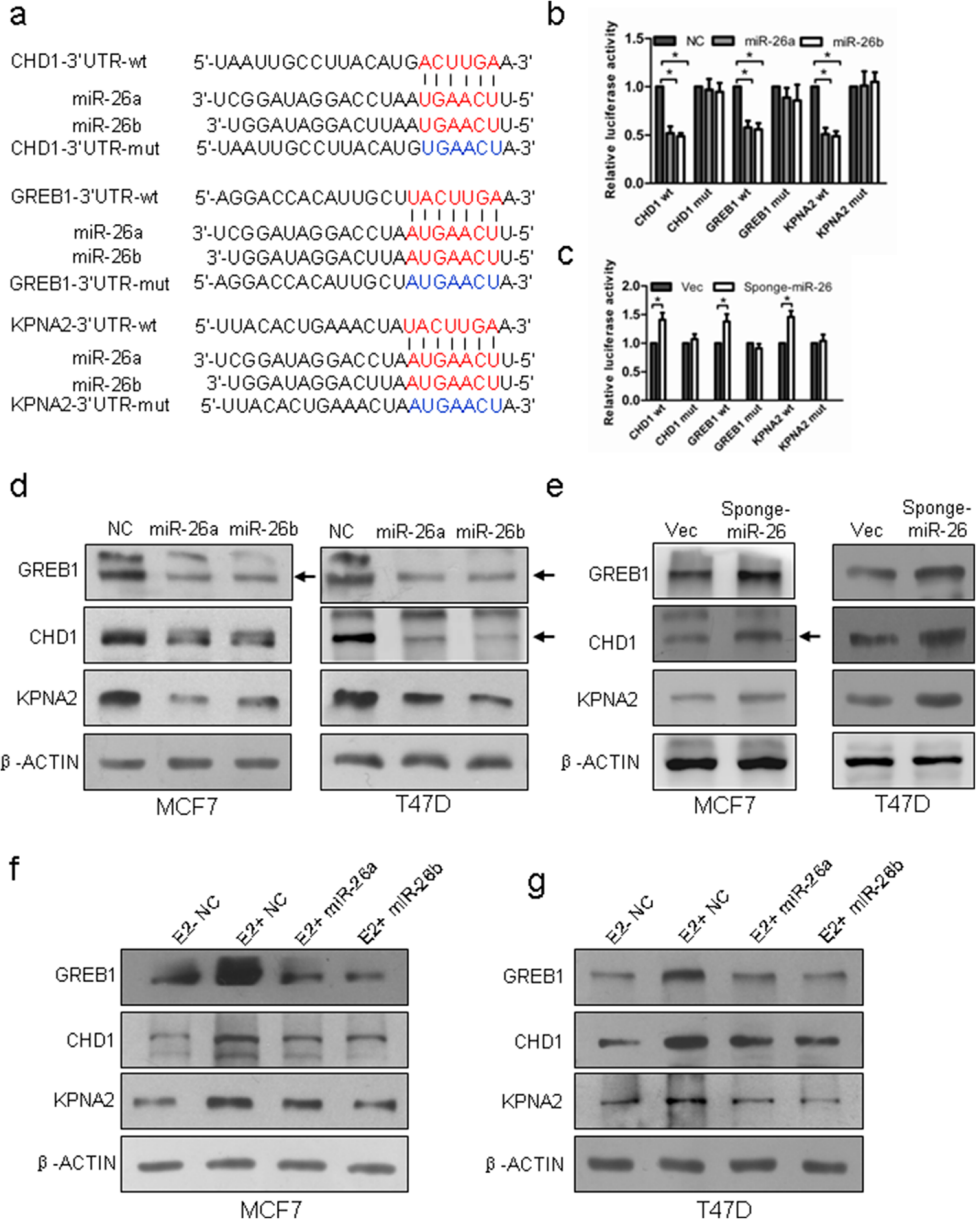
**miR-26 directly targets CHD1, GREB1 and KPNA2 genes. (a)** Putative miR-26-binding sequences in the 3′ untranslated region (UTR) of CHD1, GREB1 and KPNA2 mRNA. Mutations were generated on the CHD1, GREB1 and KPNA2 3′ UTR sequence in the complementary site for the seed region of miR-26. **(b)** Luciferase reporter assays in T47D cells, with co-transfection of wild-type (wt) or mutant (mut) 3′ UTR and microRNA (miRNA) as indicated. **(c)** Luciferase reporter assays in T47D cells, with co-transfection of wt or mut 3′ UTR and miRNA sponge as indicated. **(d)** Forced expression of miR-26a or miR-26b in MCF-7 and T47D cells decreased protein levels of CHD1, GREB1 and KPNA2 by western blot analysis. **(d, e)** After forced expression or depletion of miR-26, the expression of CHD1, GREB1 and KPNA2 protein were analyzed by western blot assay. **(f, g)** Western blotting analysis of the expression of CHD1, GREB1 and KPNA2 protein in 17β-estradiol (E2)-treated MCF-7 and T47D cells transfected with the indicated miRNA molecules. *P < 0.01.

Using TargetScan, we located the potential binding sites for miR-26 at the 3′ UTR of *CHD1*, *GREB1* and *KPNA2* and the respective mutants were further constructed (Figure 5a). We co-transfected luciferase reporter plasmids containing the 3′ UTR of each of the three genes with or without mutation and miR-26a mimics, miR-26b mimics or control oligonucleotides. Forced expression of miR-26a mimics or miR-26b mimics markedly reduced the activity of luciferase reporter genes fused to wild-type 3′ UTRs by more than 40%, but failed to affect the reporter activity when the 3′ UTR was mutated (Figure 5b). Consistently, miR-26 depletion by sponge produced a marked increase in the luciferase activity of the reporter plasmid containing the wild-type 3′ UTRs, but not their mutants (Figure 5c). miR-26 hence directly binds to the respective 3′ UTR of *CHD1*, *GREB1* or *KPNA2* to regulate their expression. We further verified that forced expression of miR-26a mimics or miR-26b mimics significantly reduced the expression of CHD1, GREB1 and KPNA2 at the protein level in MCF-7 and T47D cells (Figure 5d). As expected, the expression of CHD1, GREB1 and KPNA2 protein was increased after forced expression of Sponge-miR-26 in both MCF-7 and T47D cells (Figure 5e). Transfection of miR-26a or miR-26b mimics substantially abrogated estrogen-stimulated expression of CHD1, GREB1 and KPNA2, indicating that estrogen induced expression of CHD1, GREB1 and KPNA2 via miR-26a/b (Figure 5f,g). Immunohistochemical analysis of the tumors derived from MCF-7 cells with forced expression of miR-26 demonstrated lower expression of GREB1, CHD1 and KPNA2 compared with control tumors (Figure 2e).

### CHD1, GREB1 and KPNA2 as mediators of cell growth promoted by miR-26 depletion

**Figure 6:**
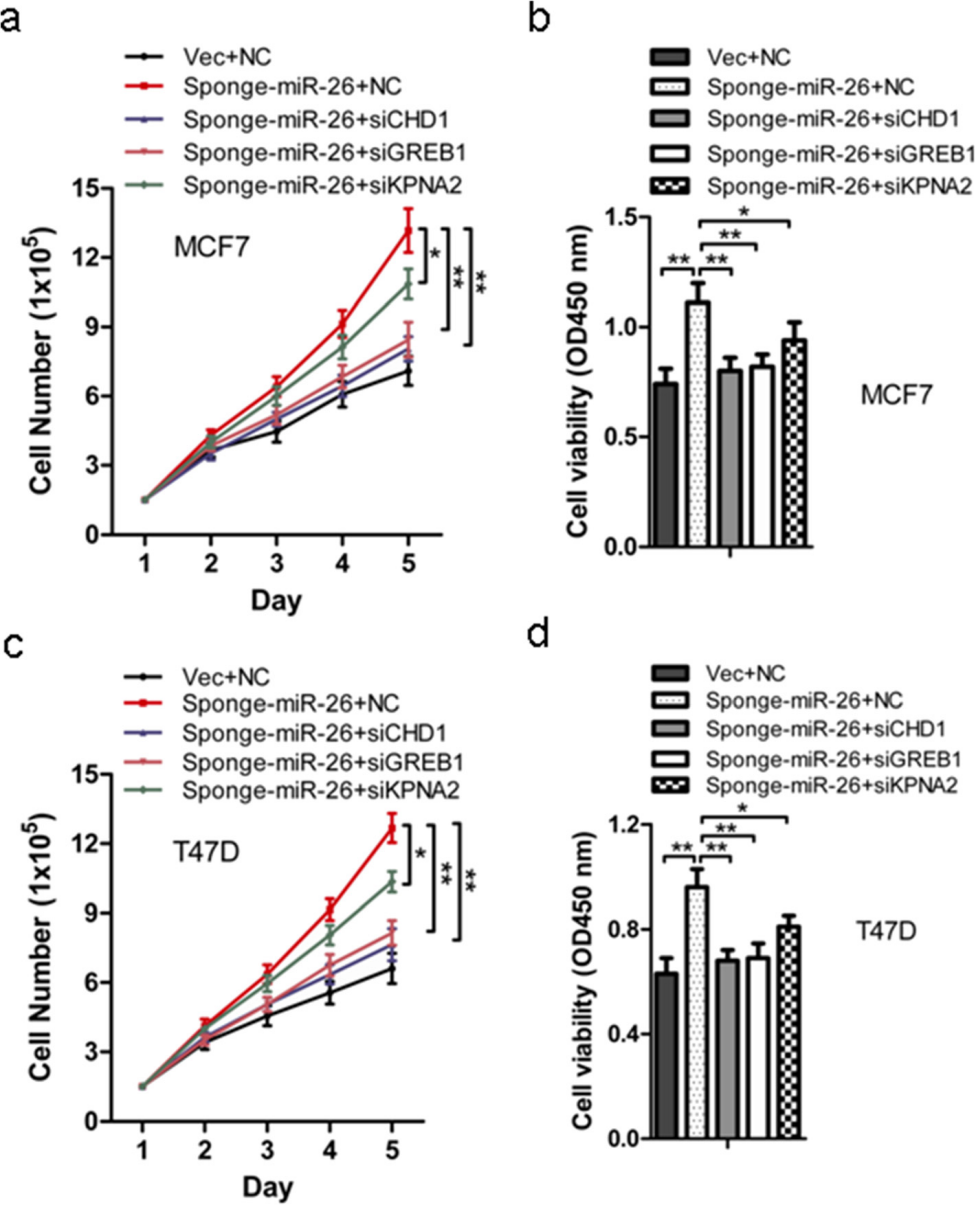
**CHD1, GREB1 or KPNA2 small interfering RNA partially mimics the effect of Sponge-miR-26 on MCF-7 and T47D cells. (a, c)** MCF-7-Vec and MCF-7-Sponge-miR-26 cells deprived of estrogen for 2 days were transfected with NC or small interfering (siRNA) and 1 day later were seeded in six-well plates without 10 nmol/l 17β-estradiol (E2). At the indicated time points after transfected NC or siRNA, MCF-7-Vec, MCF-7-Sponge-miR-26, T47D-Vec and T47D-Sponge-miR-26 cells were trypsinized and total cell numbers were counted using Trypan blue. **(b, d)** MCF-7-Vec and MCF-7-Sponge-miR-26 cells deprived of estrogen for 2 days were transfected with NC or siRNA and 1 day later were seeded in 96-well plates without 10 nmol/l E2. At 96 hours after plating, the MTT assay was performed to determine the proliferation of MCF-7-Vec, MCF-7-Sponge-miR-26, T47D-Vec and T47D-Sponge-miR-26 cells. *P < 0.05. **P < 0.01.

To further determine whether the expression of CHD1, GREB1 or KPNA2 was required for cellular events downstream of miR-26a/b, siRNAs for CHD1, GREB1 or KPNA2 were transfected into MCF-7/Vec, MCF-7/Sponge-miR-26, T47D/Vec and T47D/Sponge-miR-26 cells (Figure 6). The results showed that specific depletion of CHD1 or GREB1 or KPNA2 by siRNA significantly abrogated the enhanced growth of MCF-7 (Figure 6a,b) and T47D (Figure 6c,d) cells consequent to miR-26 depletion. The expression of CHD1, GREB1 and KPNA2 is hence required to promote cell growth consequent to miR-26 depletion.

### Estrogen-induced c-MYC expression is necessary for the repression of miR-26a and miR-26b expression

**Figure 7:**
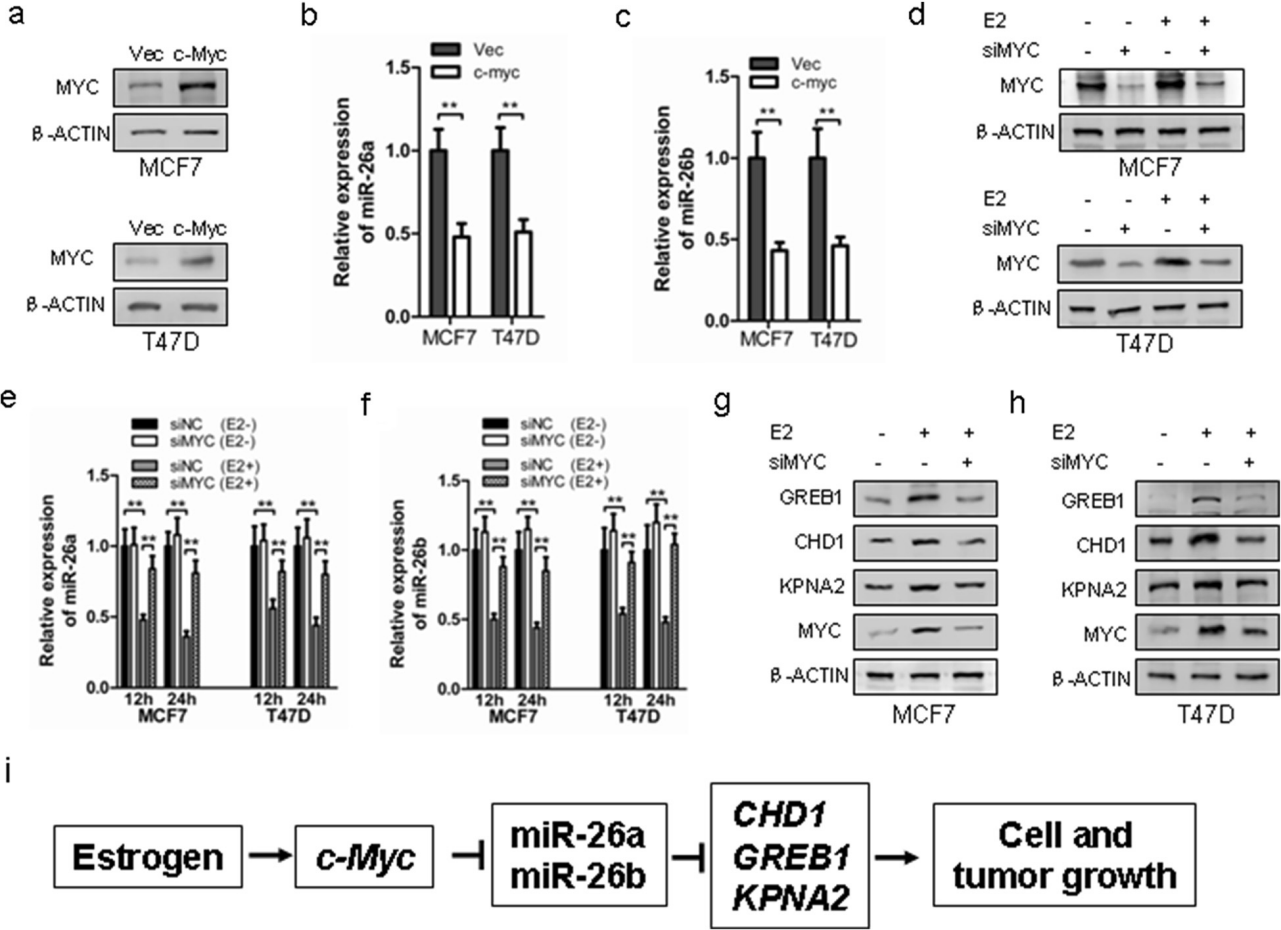
**c-MYC is required for 17β-estradiol repression of miR-26 expression. (a)** Expression of endogenous c-MYC protein in MCF-7 and T47D cells transfected with pcDNA3.1-Vec of pcDNA3.1-c-MYC. TaqMan stem-loop quantitative real-time polymerase chain reaction (qRT-PCR) analysis of the expression of miR-26a **(b)** and miR-26b **(c)** in MCF-7 and T47D cells after forced expression of c-MYC. **(d)** Expression of endogenous c-MYC protein in 17β-estradiol (E2)-treated MCF-7 and T47D cells transfected with small interfering RNA (siRNA) control or siMYC. TaqMan stem-loop qRT-PCR analysis of the expression of miR-26a **(e)** and miR-26b **(f)** in E2-treated MCF-7 and T47D cells transfected with the indicated c-MYC siRNA. U6 was used as an internal control. **(g)** Expression of endogenous CHD1, GREB1, KPNA2 and c-MYC protein in E2-treated MCF-7 cells transfected with siRNA control or siMYC. **(h)** Expression of endogenous CHD1, GREB1, KPNA2 and c-MYC protein in E2-treated T47D cells transfected with siRNA control or siMYC. β-ACTIN was used as input control. **(i)** A model of estrogen promotion of c-MYC expression, which suppressed miR-26a and miR-26b and resulted in increased expression of CHD1, GREB1 and KPNA2 and subsequent enhanced cell growth. **P < 0.01.

c-MYC was previously reported to regulate the expression of many miRNAs, including miR-26a and miR-26b [21,29,30]. To explore whether c-MYC was required for estrogen-simulated repression of miR-26a/b in MCF-7 and T47D cells, control vector or c-MYC-expressing plasmids were transfected in both cell lines. As expected, forced expression of c-MYC resulted in the reduced expression of miR-26a and miR-26b in both MCF-7 and T47D cells (Figure 7a,b,c). c-MYC has been reported as an estrogen-responsive gene and is a positive regulator of estrogen-stimulated breast cancer cell growth [31-33]. We therefore next determined whether c-MYC was involved in estrogenic suppression of miR-26a/b. We first verified the efficacy of the c-MYC-specific siRNA duplex in the presence or absence of estrogen stimulation in both MCF-7 and T47D cells (Figure 7d). Abrogation of MYC expression by siRNA resulted in a substantial increased expression of estrogen-stimulated miR-26a and miR-26b (Figure 7e,f), indicating that c-MYC was a mediator of estrogenic repression of miR-26a/b expression. We next investigated whether c-MYC expression was required for estrogen-stimulated expression of GREB1, CHD1 and KPNA2. Remarkably, depletion of MYC by siRNA significantly diminished estrogen-stimulated expression of GREB1, CHD1 and KPNA2 in both MCF-7 and T47D cells (Figure 7g,h).

### miR-26a and miR-26b expression are decreased in breast cancer

**Figure 8:**
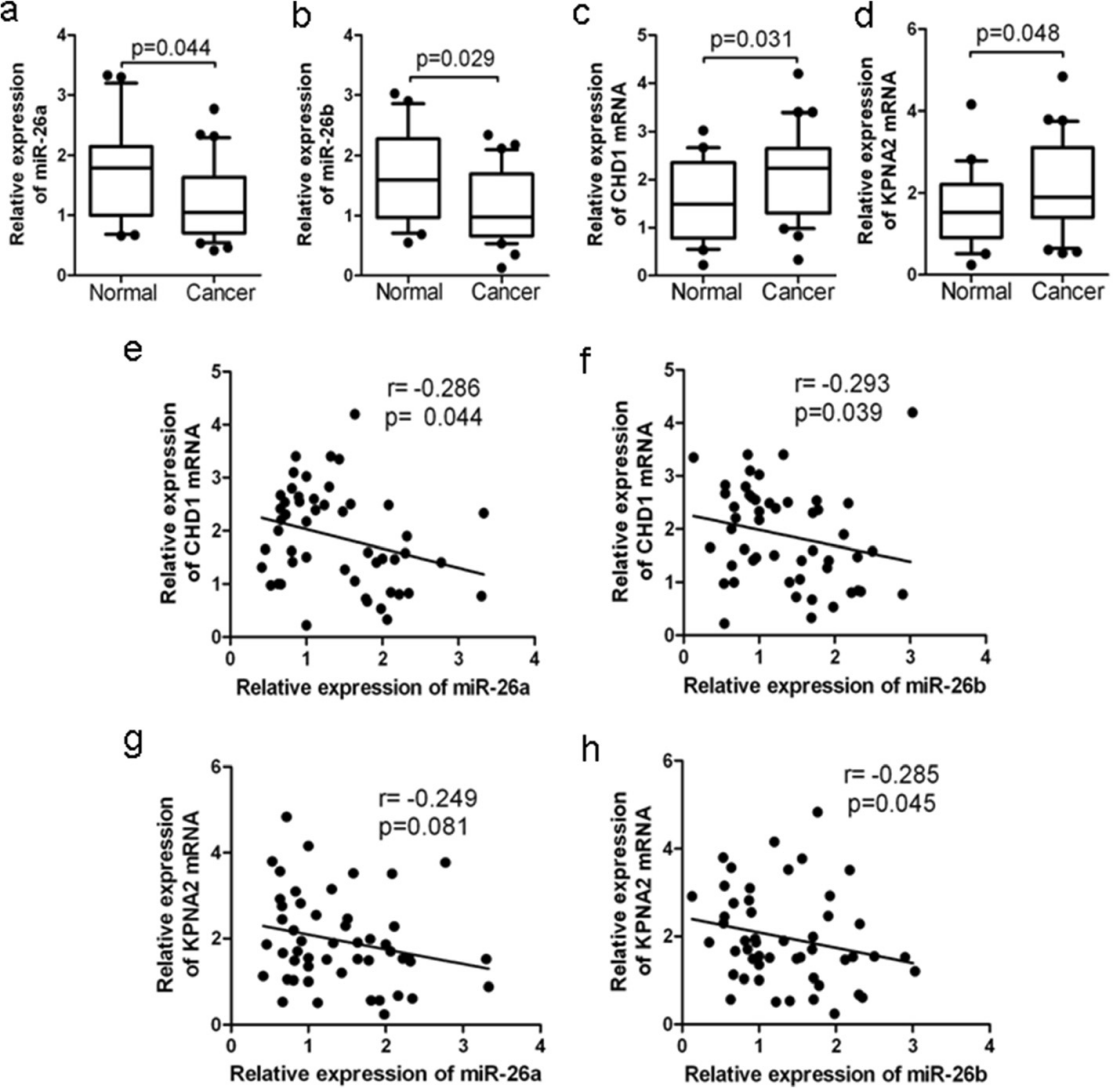
**miR-26 was downregulated in breast cancer and inversely correlated with CHD1 and KPNA2 levels. (a, b)** Relative expression levels of miR-26 in human breast specimens. MicroRNA (miRNA) levels were measured by TaqMan stem-loop quantitative real-time polymerase chain reaction. U6 was used as an internal control. **(c, d)** Relative expression levels of CHD1 and KPNA2 mRNA in breast clinical specimens. CHD1 and KPNA2 abundance was normalized to glyceraldehyde 3-phosphate dehydrogenase. **(e, f, g, h)** A statistically inverse correlation between miR-26 and CHD1 and KPNA2 mRNA levels in human breast specimens (Spearman’s correlation analysis).

We compared the levels of miR-26a/b in 20 samples of normal breast tissue and 30 ER+ breast cancer specimens by stem-loop qRT-PCR. Expression of miR-26a and miR-26b was reduced in breast cancer specimens (*P* < 0.05) compared with normal tissues (Figure 8a,b). We further evaluated the expression levels of CHD1, GREB1 and KPNA2 mRNA in these breast cancer specimens by qRT-PCR. The results showed that the average expression levels of CHD1 and KPNA2 were significantly higher in breast cancers (*P* < 0.05) than in normal breast tissues (Figure 8c,d). However, the average expression level of GREB1 was slightly lower in breast cancers (*P* > 0.05) than in normal breast tissues (Figure S7 in Additional file 1). We subsequently correlated CHD1, GREB1 and KPNA2 with miR-26 expression in these specimens. When CHD1 mRNA levels were plotted against miR-26a and miR-26b expression, significant inverse correlations were observed (*P* < 0.05; Figure 8e,f). When KPNA2 mRNA levels were plotted against miR-26b expression, significant inverse correlations were observed (*P* < 0.05; Figure 8h). However, when the KPNA2 mRNA level was plotted against miR-26a expression (*P* > 0.05, Figure 8g) or when the GREB1 mRNA level was plotted against miR-26 expression (*P* > 0.05; Figure S7 in Additional file 1), there were no significant inverse correlations.

## Discussion

Breast cancer is one of the most prevalent causes of cancer-related death for women worldwide. Estrogens play an important role in breast cancer [2,3]. Estrogen acts through ER-alpha and ER-beta, and blockade of estrogen signaling with anti-estrogens is an effective treatment for the majority of patients with ER-alpha-positive breast cancer. However, many ER+ breast cancer patients that initially respond to anti-estrogen therapy develop tumor recurrence [4]. An improved understanding of the molecular basis of estrogen treatment and the development of new strategies to increase the efficacy of anti-estrogens are required.

miRNAs have been demonstrated to play important roles in breast cancer development [5,6]. miR-26a or miR-26b were reported to be downregulated in breast cancer and they were therefore regarded as tumor suppressor miRNAs [18,19,23,34]. Estrogen treatment resulted in reduced expression of miR-26a in an ER-dependent manner [18,19]. miR-26a has also been reported to directly suppress progesterone receptor expression through binding to the progesterone receptor 3′ UTR. However, whether progesterone receptor actually mediated miR-26a repression-promoted cell growth in response to estrogen stimulation was not defined in this study [18]. Herein, we report that miR-26a and miR-26b were downregulated following estrogen treatment in ER+ human breast cancer cells. To date, experimentally validated miR-26 targets contain many oncogenes, including *EZH2*[30], *CCND2*[21], *CCNE2*[21], *MTDH*[34] and *SLC7A11*[35]. However, none of these were reported to be estrogen responsive.

To determine how the altered expression of miR-26 may mediate estrogenic effects, we first screened estrogen-responsive genes that were also predicted to be miR-26 target genes by both bioinformatics and RT-PCR analysis. *GREB1* and nine other genes (*AGPAT5*, *AMMECR1*, *CHD1*, *ERLIN1*, *HSPA8*, *KPNA2*, *MREG*, *NARG1* and *PLOD2*) were subsequently identified. Further verification by luciferase reporter assay identified nine other genes (*AGPAT5*, *CHD1*, *ERLIN1*, *GREB1*, *HSPA8*, *KPNA2*, *MREG*, *NARG1* and *PLOD2*) that were directly targeted by miR-26 via their 3′ UTR. Functional screening suggested that only three estrogen-regulated miR-26 target genes (*CHD1*, *GREB1* and *KPNA2*) were involved in the regulation of estrogen-promoted cell proliferation. We thus identified CHD1, GREB1 and KPNA2 as novel functional targets of miR-26. The expression of miR-26a and miR-26b in human breast cancer cells suppressed cell growth, at least partly through repression of CHD1, GREB1 and KPNA2. Consistently, depletion of CHD1, GREB1 or KPNA2 produced cell growth inhibition similar to the phenotypes induced by miR-26a or miR-26b restoration. These data verified previous findings, in which the depletion of GREB1 suppressed MCF-7 cell proliferation [25]. However, we further determined that increased expression of CHD1, GREB1 and KPNA2 following estrogen treatment was partially due to the decreased expression of miR-26a and miR-26b. Moreover, we determined that CHD1 and KPNA2 were necessary for estrogen-stimulated proliferation of breast cancer cells.

c-MYC, as a well-known estrogen-responsive gene, has been shown to mediate the proliferative effects of estrogen in ER+ breast cancer cells [36]. Antisense oligonucleotides directed against c-MYC inhibit estrogen-induced cell proliferation in a manner similar to that of anti-estrogens [37]. In this study, we examined the possibility of the estrogenic effects of c-MYC mediating a c-MYC-regulated miRNA network. c-MYC has been reported to suppress the expression of many miRNAs, including miR-26a and 26b [21,29,30]. However, it was unclear whether c-MYC is involved in estrogenic repression of miR-26a and miR-26b expression. Our observations imply that c-MYC is necessary for inhibition of miR-26a and miR-26b expression produced by estrogen. The results further suggest that aberrant c-MYC expression, which is frequently observed in human breast cancers [38], can contribute to the estrogenic effect by alteration of miR-26a and miR-26b expression. Our results have thus identified a novel pathway by which estrogen utilizes c-MYC for suppression of miR-26a/b to promote the expression of CHD1, GREB1 and KPNA2 (Figure 7i).

This study systematically investigated the functionality and mechanism of miR-26a and miR-26b in estrogen-promoted ER+ breast cancer cell proliferation. GREB1, CHD1 and KPNA2 were identified as novel targets of miR-26a/b and were demonstrated to be necessary for estrogen-promoted ER+ breast cancer cell proliferation. Further, we demonstrated that estrogen-stimulated c-MYC expression was both sufficient and necessary for the diminished expression of miR-26a and miR-26b. These findings will facilitate a better understanding of the molecular pathogenesis of breast cancer and suggest that mimics of miR-26a and miR-26b may be considered as a novel strategy for breast cancer therapy.

## Conclusions

Estrogen signaling is pivotal in the progression of ER+ breast cancer. An improved understanding of the molecular basis of estrogen action and the development of new strategies to improve the efficacy of anti-estrogens are required. We have therefore identified a novel estrogen/MYC/miR-26 axis that mediated estrogen stimulated cell growth via CHD1, GREB1 and KPNA2 and suggest that upregulation of miR-26a and miR-26b may be considered as novel strategy for breast cancer therapy.

## Abbreviations

E2: 17β-estradiol; ER: estrogen receptor; miRNA: microRNA; PCR: polymerase chain reaction; RT: real-time; siRNA: small interfering RNA; UTR: untranslated region.

## Competing interests

The authors declare that they have no competing interests.

## Authors’ contributions

ST maintained all of the cell cultures, designed the siRNA experiment, ran qRT-PCR and western blots, performed the *in vivo* study, was involved in the design of the study and critically revised the manuscript. KD provided all of the plasmids used in the study, performed the siRNA experiment, ran qRT-PCR and western blots, performed the luciferase assays, participated in the design of the study and critically revised the manuscript. RL and WZ performed immunohistochemistry assays, were involved in the RNA and protein study, and participated in the manuscript writing. GL participated in the animal study and critically revised the manuscript. XK and PQ participated in data collection and were involved in the manuscript writing. PEL participated in data analysis and provided critical review of the manuscript. TZ conceived of the ideas of the manuscript, wrote the manuscript. All authors approved the final manuscript for publication.

## Supplementary Material

Additional file 1 **contains the following additional data. Figure S1** shows a heat-map of 10 miRNAs differentially expressed between MCF7/estrogen-deprived and MCF7/estrogen-treated cells. **Figure S2** shows miR-26-inhibited breast cancer cell growth: **(a)** total cell number of T47D was counted; **(b)** MTT assay was performed to determine the proliferation of T47D cells; **(c)** T47D-Vec and T47D-Sponge-miR-26 cells were trypsinized and total cell number was counted; **(d)** MTT assay was performed to determine the proliferation of T47D-Vec and T47D-Sponge-miR-26 cells; **(e, f)** MDA-MB-231 and BT549 cells were transfected with NC, miR-26a or miR-26b mimics and total cell number was counted; **(g, h)** MTT assay was performed to determine the proliferation of MDA-MB-231 and BT549 cells. **Figure S3** shows regulation of gene expression by miR-26: ectopic expression of miR-26b in T47D cells decreased mRNA levels of the indicated 26 genes. **Figure S4** shows promotion of gene expression by E2: **(a)** MCF-7 and **(b)** T47D cells were treated with 10 nmol/l E2 and mRNAs were isolated; mRNA level measured by qRT-PCR. **Figure S5** shows efficacy and specificity of siRNA duplexes: ectopic expression of siRNA in MCF-7 cells decreased mRNA levels of the indicated 10 genes by qRT-PCR. **Figure S6** shows that siRNA-mediated depletion of AGPAT5, ERLIN1, HSPA8, MREG, NARG1 or PLOD2 expression was required for the tumor suppressor functions of miR-26: **(a, c)** MCF-7 or T47D cells deprived of estrogen for 2 days were transfected with NC or siRNA, and total cell number was counted; **(b, d)** MCF-7 or T47D cells deprived of estrogen for 2 days were infected with NC or siRNA, and MTT assay was performed to determine the cell proliferation. **Figure S7** shows **(a)** relative expression level of GREB1 in breast human specimens and **(b, c)** a statistical correlation between miR-26 and GREB1 mRNA levels in human breast specimens (Spearman’s correlation analysis). *P < 0.05. **P < 0.01. Click here for file
