# Supplementary figures and images for: Identification of miR-26 as a key mediator of estrogen stimulated cell proliferation by targeting CHD1, GREB1 and KPNA2
Source: Breast Cancer Res. 2014 Apr 15;16(2):R40. doi: 10.1186/bcr3644 (PMC4053242; doi:10.1186/bcr3644)

Figure S1

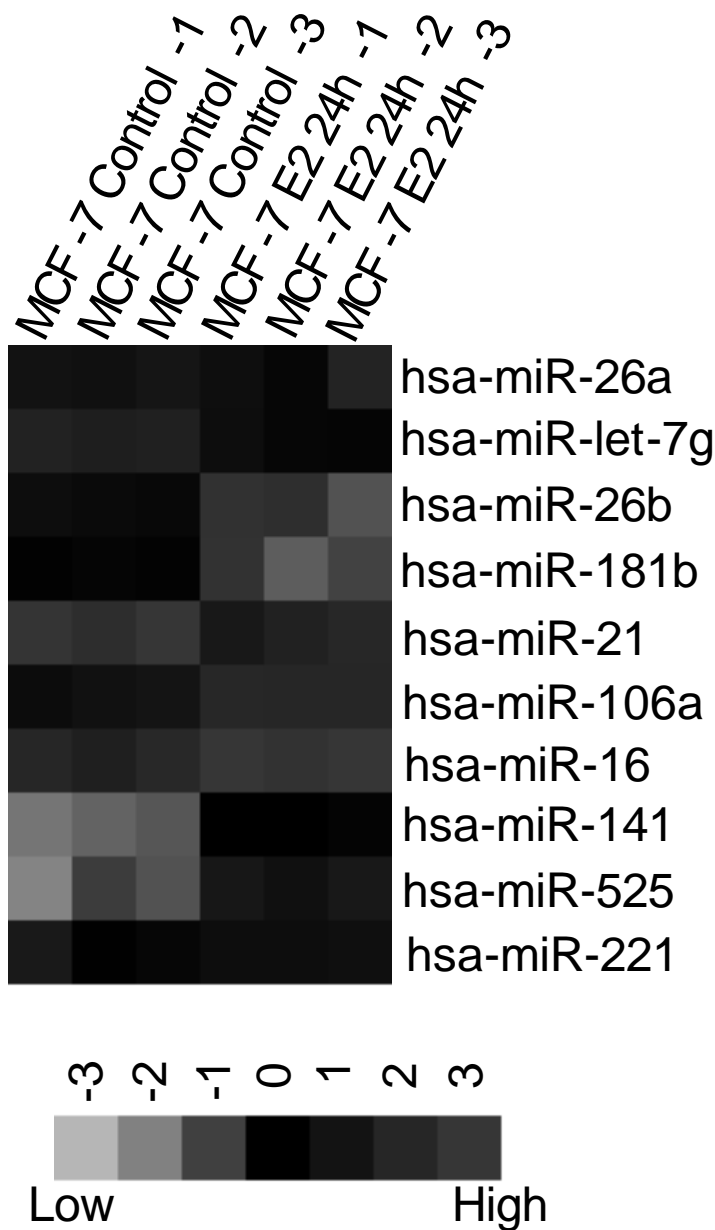

Figure S2

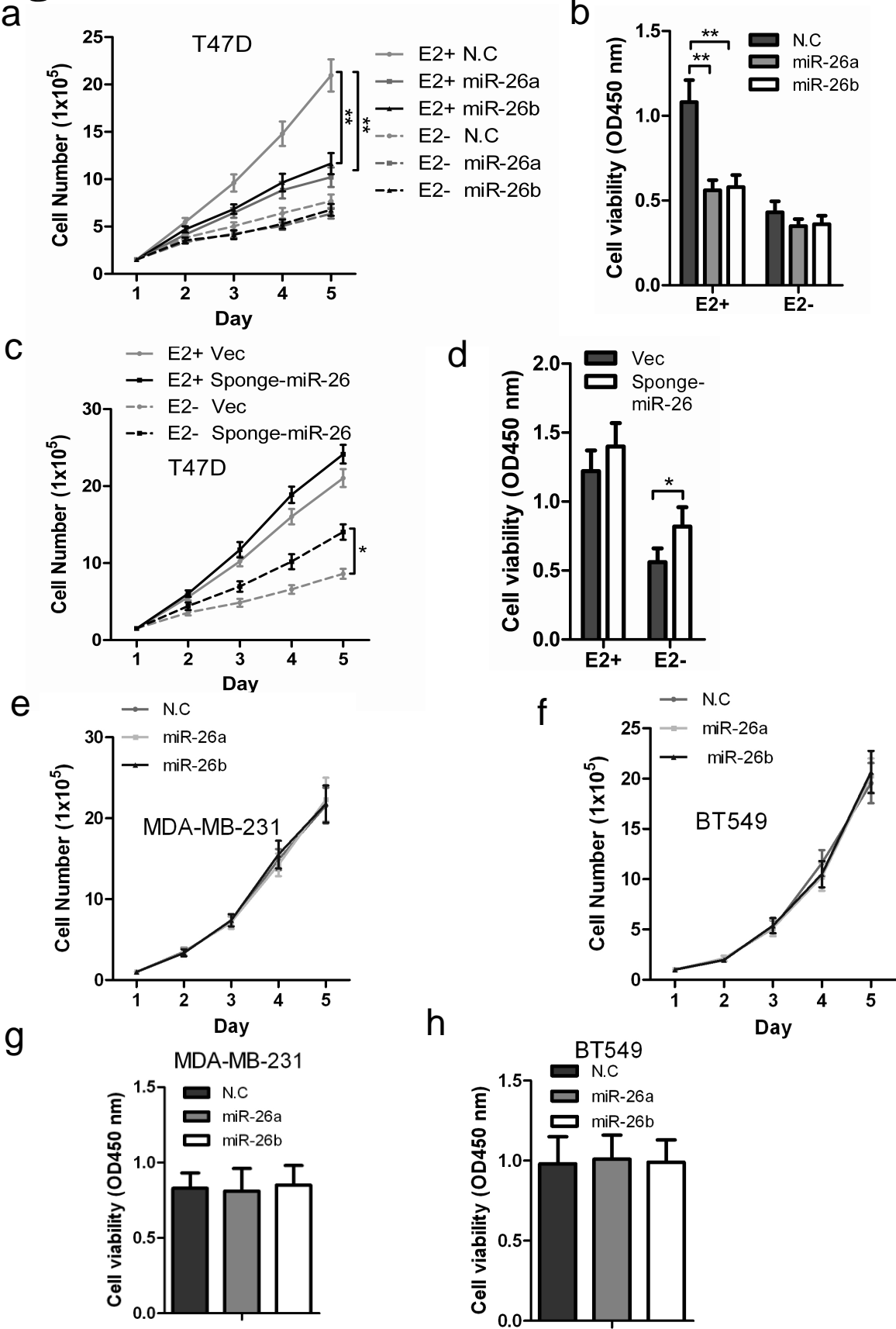

Figure S3

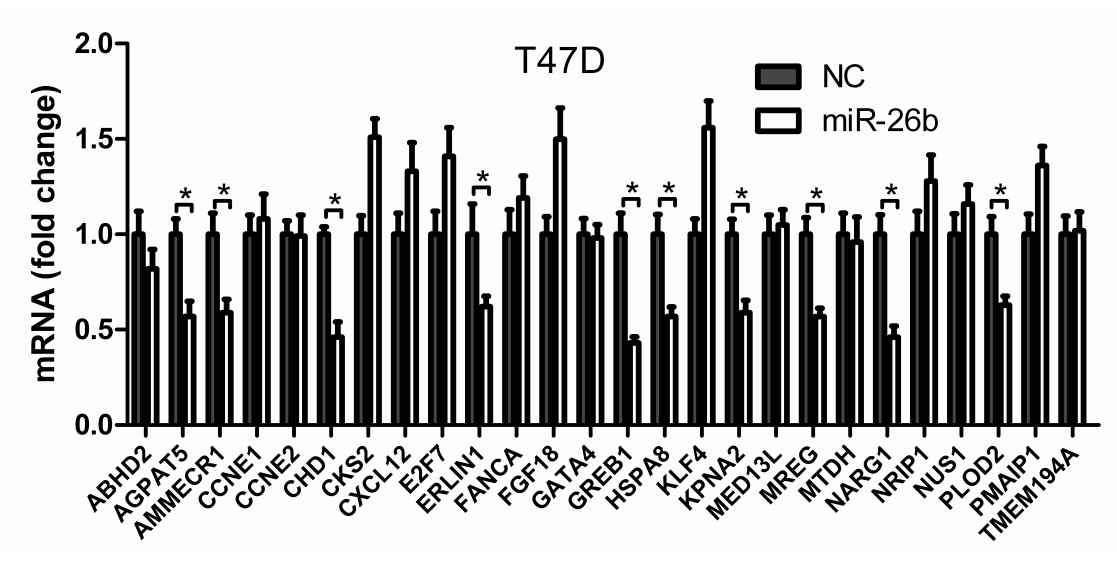

Figure S4

a

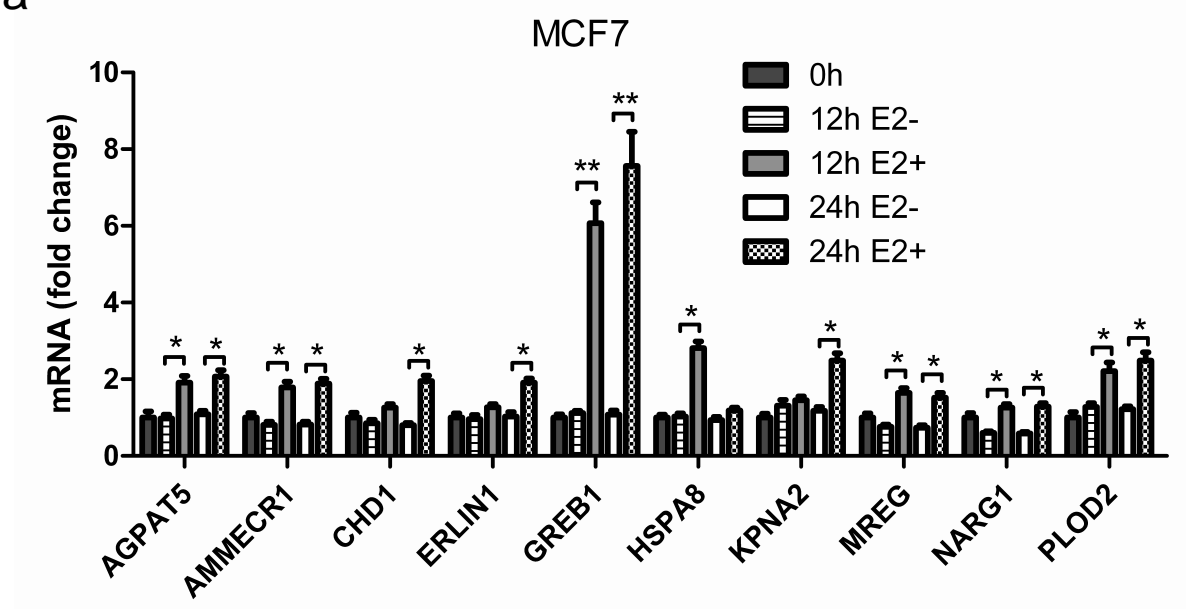

b

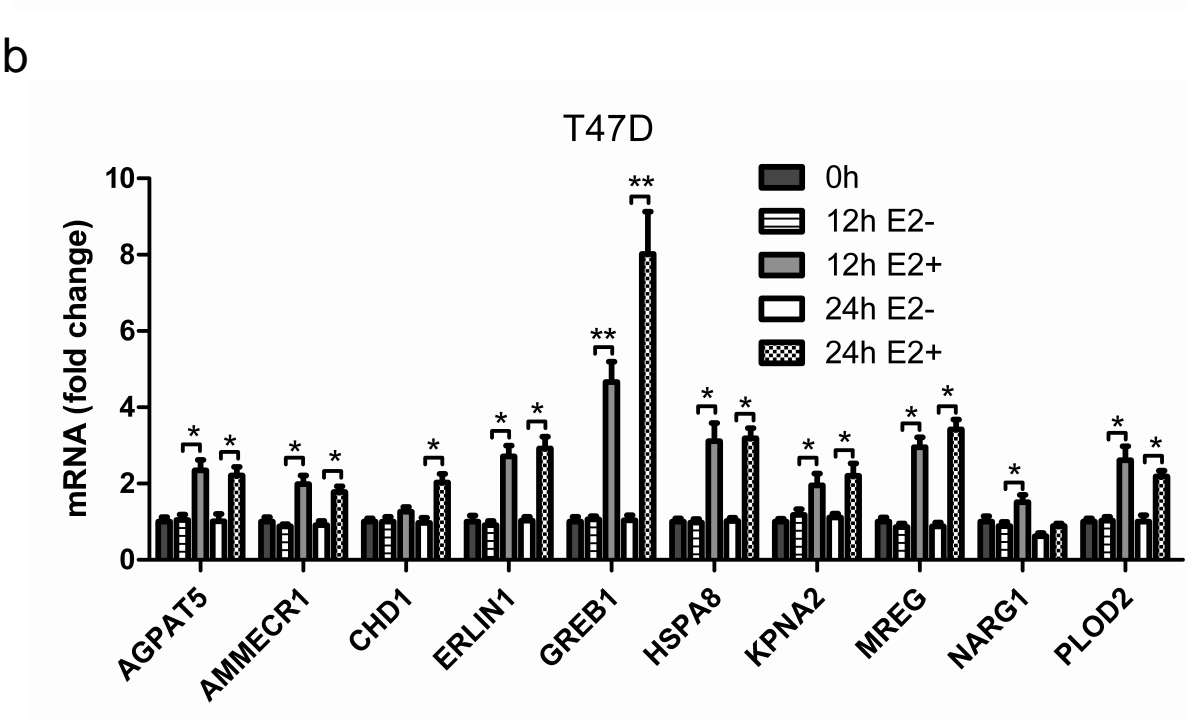

Figure S5

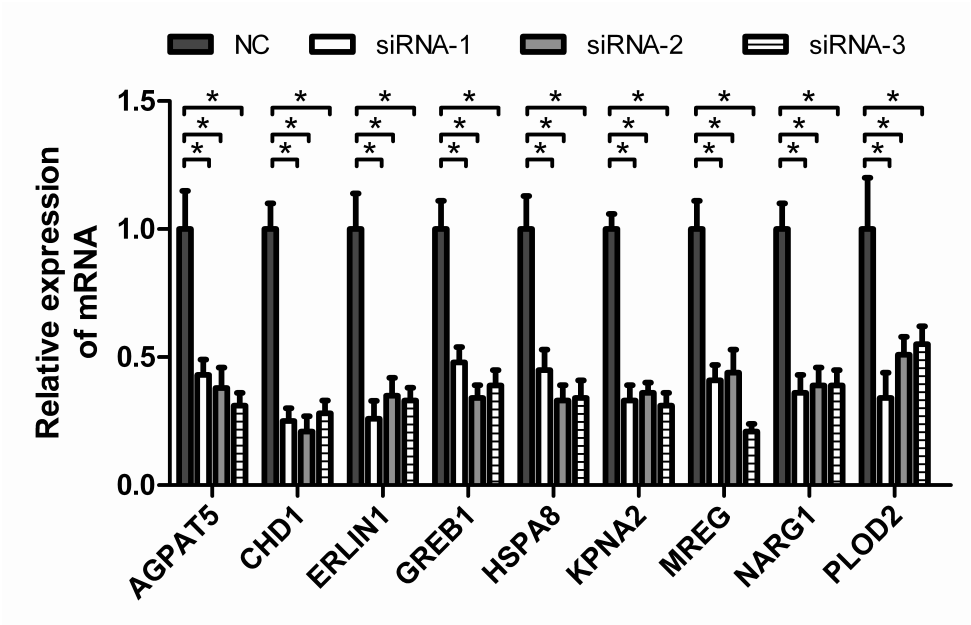

## Figure S6

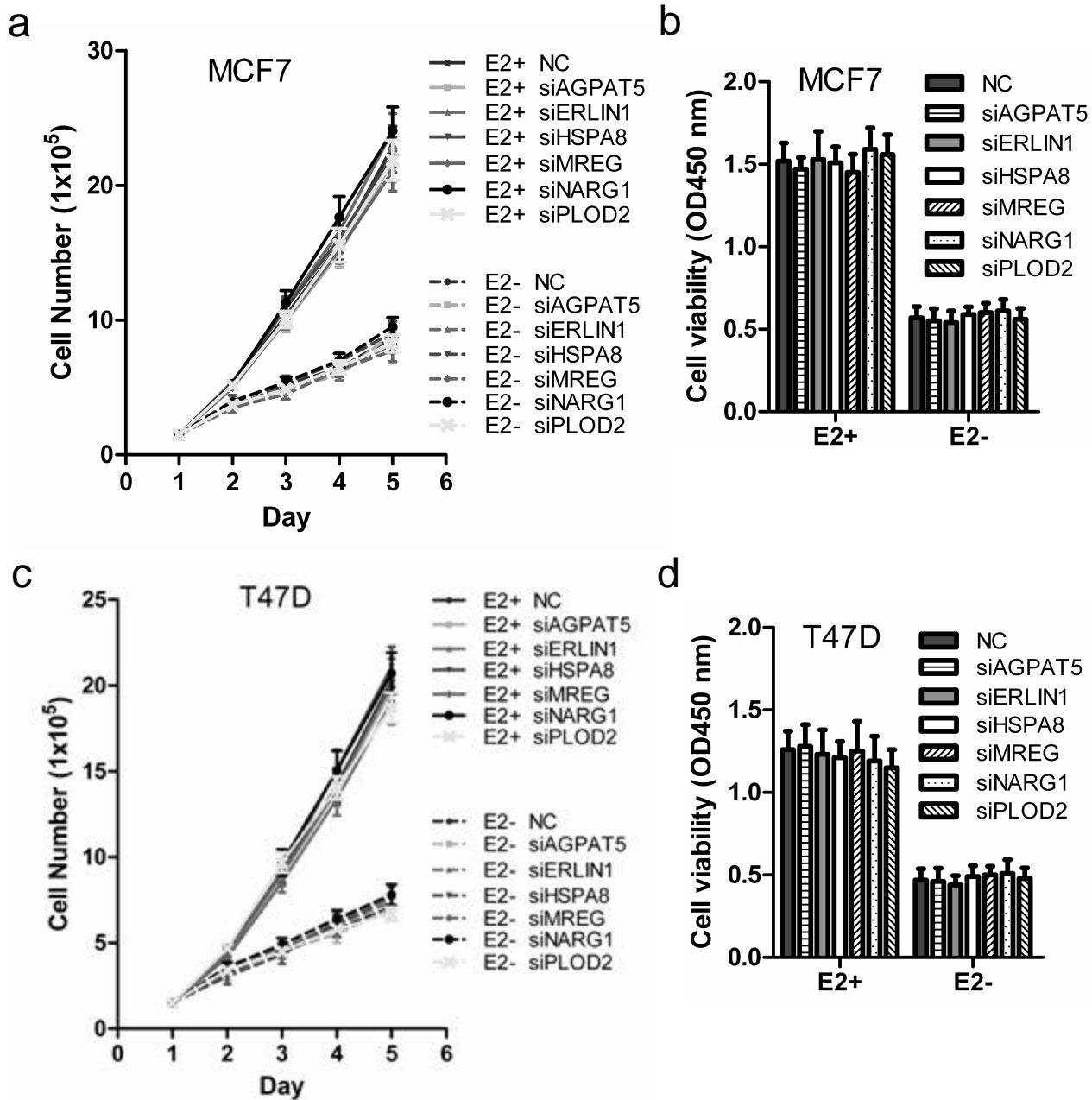

Figure S7

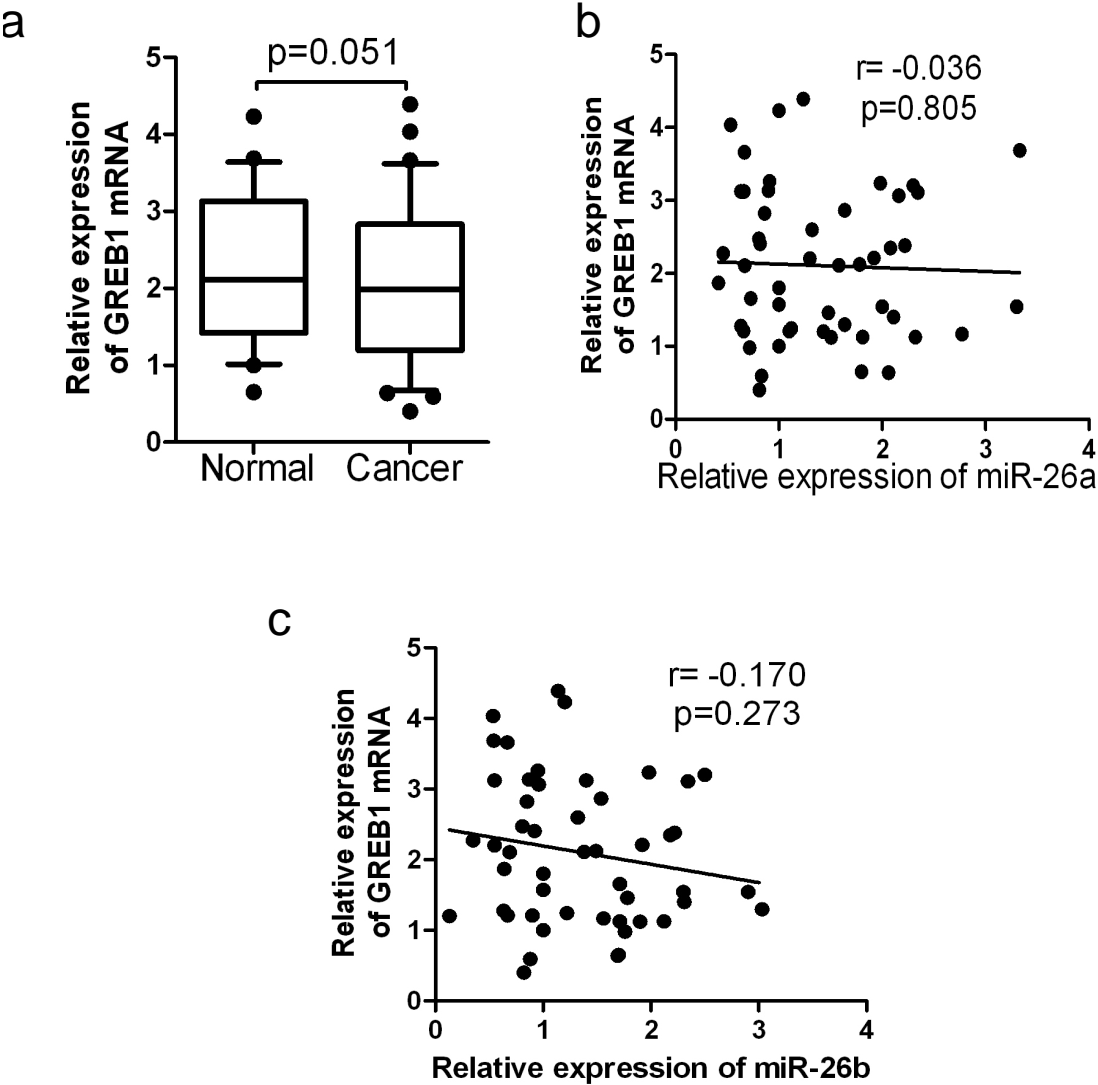

Supplement: Additional file 1 — contains the following additional data. Figure S1 shows a heat-map of 10 miRNAs differentially expressed between MCF7/estrogen-deprived and MCF7/estrogen-treated cells. Figure S2 shows miR-26-inhibited breast cancer cell growth: (a) total cell number of T47D was counted; (b) MTT assay was performed to determine the proliferation of T47D cells; (c) T47D-Vec and T47D-Sponge-miR-26 cells were trypsinized and total cell number was counted; (d) MTT assay was performed to determine the proliferation of T47D-Vec and T47D-Sponge-miR-26 cells; (e, f) MDA-MB-231 and BT549 cells were transfected with NC, miR-26a or miR-26b mimics and total cell number was counted; (g, h) MTT assay was performed to determine the proliferation of MDA-MB-231 and BT549 cells. Figure S3 shows regulation of gene expression by miR-26: ectopic expression of miR-26b in T47D cells decreased mRNA levels of the indicated 26 genes. Figure S4 shows promotion of gene expression by E2: (a) MCF-7 and (b) T47D cells were treated with 10 nmol/l E2 and mRNAs were isolated; mRNA level measured by qRT-PCR. Figure S5 shows efficacy and specificity of siRNA duplexes: ectopic expression of siRNA in MCF-7 cells decreased mRNA levels of the indicated 10 genes by qRT-PCR. Figure S6 shows that siRNA-mediated depletion of AGPAT5, ERLIN1, HSPA8, MREG, NARG1 or PLOD2 expression was required for the tumor suppressor functions of miR-26: (a, c) MCF-7 or T47D cells deprived of estrogen for 2 days were transfected with NC or siRNA, and total cell number was counted; (b, d) MCF-7 or T47D cells deprived of estrogen for 2 days were infected with NC or siRNA, and MTT assay was performed to determine the cell proliferation. Figure S7 shows (a) relative expression level of GREB1 in breast human specimens and (b, c) a statistical correlation between miR-26 and GREB1 mRNA levels in human breast specimens (Spearman’s correlation analysis). *P < 0.05. **P < 0.01. [file bcr3644-S1.pdf]
